# The seeds of tomorrow: SEAD reference panel promotes genomics in Asian human populations

**DOI:** 10.3389/fgene.2026.1926569

**Published:** 2026-10-29

**Authors:** Lidiya Ashenafi, David Karasik

**Affiliations:** 1 The Musculoskeletal Genetics Laboratory, The Azrieli Faculty of Medicine, Bar-Ilan University, Safed, Israel; 2 Hebrew SeniorLife, Hinda and Arthur Marcus Institute for Aging Research, Boston, MA, United States

**Keywords:** Asian ancestry, bone mineral density, genome-wide association study, rare variants, SNTG1 gene

## Abstract

Success of a genome-wide association study (GWAS) depends on the density of variants available for analysis; these variants are sometimes rare and ancestry-specific. Imputation of un-genotyped variants is used to boost the resolution and power of GWAS; however, majority of large imputation reference panels do not adequately reflect the populations of Asia, which comprise approximately 60% of the world’s population. South and east Asian reference database (SEAD) addresses this gap by providing a large genetic reference panel derived from more than 11,000 individuals across 17 Asian countries. Here we explain how the SEAD reference panel fits into the larger group of tools for GWAS imputation, with regard of population representation, rare-variant interpretation, functional validation of identified gene, and discuss potential future developments essential to enhance genomic coverage.

## Introduction

A genome-wide association study (GWAS) is a method used to identify genomic variants that are linked to disease or a trait (Institute, 2025). The performance of GWAS improves as the density of variants available for analysis increases. Imputation enhances this density by filling in untyped genetic variants from array or low-coverage data, thereby increasing the number of variants that can be analyzed and boosting the statistical power of GWAS (Berdnikova et al., 2024). However, majority of large imputation reference panels such as 1000 genomes (1KGP) and Trans-Omics for Precision Medicine (TOPMed), are skewed toward European ancestries, making imputation lacks fairness (Cahoon et al., 2024). This gap highlights the need for ancestry-specific appropriate reference panels. South and east Asian reference database (SEAD) address this need by providing a large genetic reference panel of over 22,000 haplotypes (two per individual) derived from more than 11,000 individuals across 11 Asian countries (and five different ethnicities from India) (Yang et al., 2024). The following reflection will explain how the SEAD reference panel fits into the larger group of tools for genotype imputation, critically evaluates its strengths and limitations in the context of population representation, GWAS trait and population selection, population, rare-variant interpretation, and discusses its potential future development.

## Population representation and coverage in SEAD

SEAD was constructed by integrating existing multi-population panels (Westlake BioBank for Chinese (WBBC), SG10K, GAsP, and 1KGP), thus it increased both reference haplotype size and geographic span relative to the individual component panels and improved the imputation of rare and low-frequency variants in some of the evaluated Asian populations. While earlier efforts also combined reference panels for Asian populations (Chou et al., 2016; Choi et al., 2023), SEAD achieved greater population representation with especially strong coverage of individuals from China, Singapore, and India, increased both reference haplotype size and geographic span relative to other Asia based panes and improved the imputation of particularly rare and low-frequency variants in several Asian populations. Despite including samples from 11 Asian countries (17 ethnicities; from Yang et al., 2024), substantial gaps remain, with several populations, such as, Taiwanese, Myanmar, and others in East Asia, as well as Bhutan, the Maldives, Nepal, and others in South Asia, still absent or poorly represented in existing reference resources. Additionally, the sample distribution is also skewed, with China and Singapore contributing more than 80% of all individuals (Figure 1A), whereas many other East and South Asian populations contribute substantially fewer haplotypes. Thus, the geographic coverage of the panel should not be interpreted as equivalent representation across Asia, particularly because rare variant imputation depends strongly on the number of ancestry-matched haplotypes available for the target population.

**FIGURE 1:**
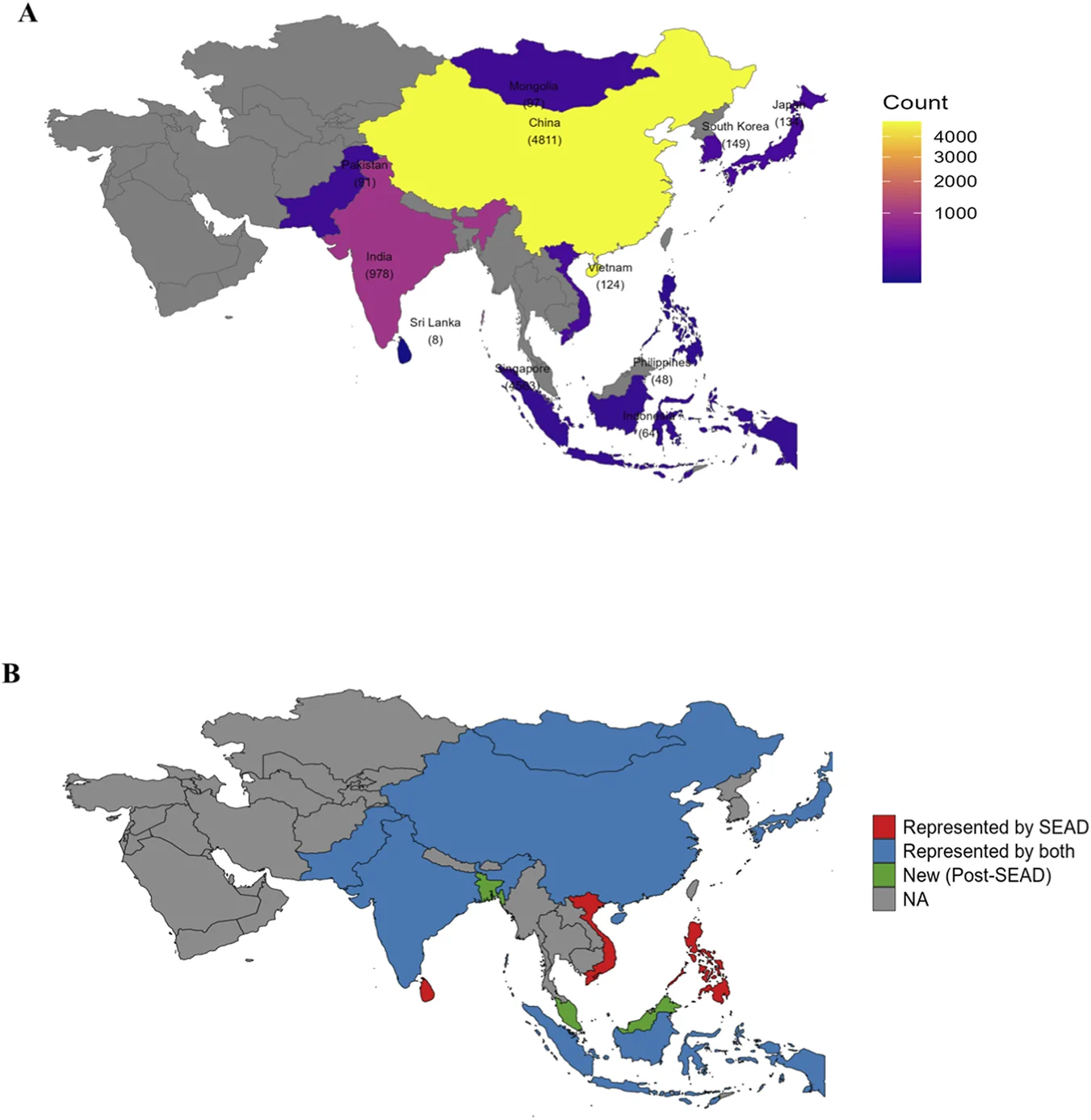
Geographic representation of Asian populations included in reference panels. **(A)** Countries represented in the SEAD reference panel. Color intensity reflects the number of individuals contributed from each country, with yellow indicating higher representation and purple indicating lower representation. **(B)** Countries represented exclusively by SEAD and those additionally captured by newly available post-SEAD biobanks. Distinct colors indicate populations represented by SEAD alone, new biobanks alone, or both. Geographical boundaries were obtained from Natural Earth using the R package rnaturalearth. Population and reference‐panel information were compiled from the SEAD study (Yang et al., 2024) and other published Asian genomic reference‐panel studies cited in the manuscript.

While a few new Asian reference panels have been introduced since SEAD, most still focus on populations already well represented such as those from China, Japan, and India, still offering limited expansion of geographic coverage (Figure 1B) (Flanagan et al., 2024; Cengnata et al., 2024; Yu et al., 2023; Li et al., 2026). Overall, Han Chinese, Japanese, and Korean populations are among the most extensively represented Asian groups in genotype-imputation resources, with both large population-specific panels and inclusion in broader regional or global panels. Han Chinese are represented by resources such as ChinaMAP, NyuWa, WBBC, and CKB (Yu et al., 2023; Li et al., 2021; Zhang et al., 2021; Cong et al., 2022), as well as 1KGP, GenomeAsia, SG10K, and NARD (Auton et al., 2015; Yoo et al., 2019; GenomeAsia100K Consortium, 2019; Wu et al., 2019). Japanese populations are represented through 1KJPN, jMorp/TMM, and BioBank Japan 1 KG+7K, and JHRP (Flanagan et al., 2024; Kawai et al., 2015; Tadaka et al., 2024; Higasa et al., 2026), while Korean populations are covered by Korea1K, Korea4K, KRG (Jeon et al., 2020; Jeon et al., 2024; Hwang et al., 2022), and broader Northeast Asian panels. South Asian representation has also improved through GAsP2 and LASI-DAD (Wall et al., 2023; Li et al., 2026), particularly for Indian, Pakistani, and Bangladeshi populations, together with representation in 1KGP, GenomeAsia, and SG10K (Auton et al., 2015; GenomeAsia100K Consortium, 2019; Wu et al., 2019). In contrast, many Central Asian, Himalayan, and Indigenous or minority populations from Southeast Asia remain poorly represented in both population specific and broad Asian or global panels.

## Imputation accuracy for common and rare variants

SEAD demonstrated improved imputation particularly for rare and low frequency variants relative to the evaluated 1KGP, TOPMed and ChinaMAP panels in the selected East and South Asian datasets. For most South and Central Asian populations evaluated in the original study, it outperforms imputation with TOPMed (Taliun et al., 2021), 1KGP, and ChinaMAP. However, these comparators comprise two global panels and one Chinese specific panel and did not benchmark SEAD against a large, dedicated South Asian reference resource, such as GAsP2 (Wall et al., 2023). In East Asian populations, SEAD outperformed TOPMed and 1KGP but did not consistently outperform ChinaMAP, which showed higher accuracy in Han Chinese samples, whereas SEAD showed similar performance in Japanese samples and better performance in Cambodian samples (Yang et al., 2024). These findings suggest that the benefit of a reference panel depends on both panel size and genetic similarity to the target population. Asian enriched panels outperform poorly ancestry matched global panels, but one broad Asian panel was not shown to be universally better than intensely sampled population specific panels. When we came to common variants the evidence for improved common variant imputation using SEAD is less conclusive than that for rare and low-frequency variants. The panel performance analyses were primarily restricted to variants with minor allele frequency (MAF) below 5%, with the highest reported frequency bin being 2%–5%; therefore, common variants with MAF of at least 5% were not evaluated separately (Yang et al., 2024).

Given these results and considering the genetic distance between the EAS and SAS populations (a recent study based on 1KGP samples reported the F -statistics (*F*_*ST*_) between SAS and EAS is 0.06) (Levi et al., 2024), population specific rather than broadly continental reference panels and fair representation from both groups are likely to improve imputation accuracy for rare variants particularly.

## Eyes on the prize: GWAS trait and population selection

The researchers performed GWAS on two correlated bone mineral density (BMD) traits (Hip and Femoral neck (FN)) from WBBC collection using variants imputed by SEAD. Incorporating other BMD traits, such as lumbar spine BMD could further strengthen the study; this trait is available in the WBBC dataset as well (Zhu et al., 2021) has high heritability (approximately 50%–80%) and reflects trabecular bone, which may capture additional site-specific genetic signals (Piroska et al., 2021). Including additional Asian populations in the GWAS would also be valuable because reduced BMD is not a phenomenon unique to Chinese individuals; similar patterns have been reported in Japanese, Korean, Indian, and Pakistani populations (Lo et al., 2023; Lo et al., 2025; Sng et al., 2024). This is particularly important given the population-specificity of rare variants, which may lead to genetic associations that are unique to some Chinese subgroups and may not generalize to other Asian or global populations.

## Balance between common and rare variant genome-wide analyses

In their GWAS, the researchers (Yang et al., 2024) identified both common and rare variants associated with the BMD traits after imputing genotype data with SEAD. Although the study primarily emphasized the added value of SEAD for rare-variant analysis, common variants remain important because they account for a substantial proportion of the associations detected in conventional GWAS (Sng et al., 2024). Recent BMD studies similarly show that common variants contribute substantially to BMD-related genetic variation and constitute the majority of loci identified by large-scale GWAS, whereas rare variants generally explain a smaller proportion of the observed associations (Patiño-Salazar et al., 2025; Wainschtein et al., 2026). Consistent with this, the SEAD study identified common-variant associations at *FMN2* and near *SOX4*, both of which had previously been reported for heel, total-body, or femoral-neck BMD, thereby providing confirmation of established BMD loci. Beyond these previously identified common-variant associations, the study reported a previously unreported rare-variant association near *Syntrophin gamma 1* (*SNTG1*) for hip BMD. Importantly, this signal was not unique to SEAD imputation: the same lead variant was also recovered after imputation with ChinaMAP, WBBC-seq, and SG10K, with comparable allele frequencies, effect estimates, and genotype counts.

## Allele frequency patterns at the *SNTG1* locus

In their Yang et al. (2024) the authors provide the frequency distribution of the rare signals identified at the *SNTG1* locus. They report that these variants are rare in Non-Finnish European and South Asian populations, whereas they are more common in African American populations, highlighting the population-specific patterns at this locus. We plotted the variants identified in this study using the data provided in Figure 2. Differences in allele frequency and ancestry-specific linkage disequilibrium (LD) patterns can affect imputation accuracy, particularly for rare variants, and can subsequently influence statistical power and the ability to detect or replicate association signals across populations. A previous study conducted on Hispanic cohort also reported *SNTG1* locus associated with osteoporosis, although through a different low-frequency variant, rs72645876 (Fouhy et al., 2024). So, the significance of the *SNTG1* finding lies in improved detectability of a rare variant signal in an ancestry-matched cohort, rather than demonstrating that *SNTG1* variation is exclusive to East Asian population. In addition, the Musculoskeletal Knowledge Portal (MSK-KP) database reports that common genetic variants in this gene locus are linked to heel bone density in the Europeans (p = 1.76e-6). Although this association did not reach genome-wide significance, it provides additional locus-level evidence that variation (rare or common) in the *SNTG1* region is relevant to bone-related phenotypes.

**FIGURE 2:**
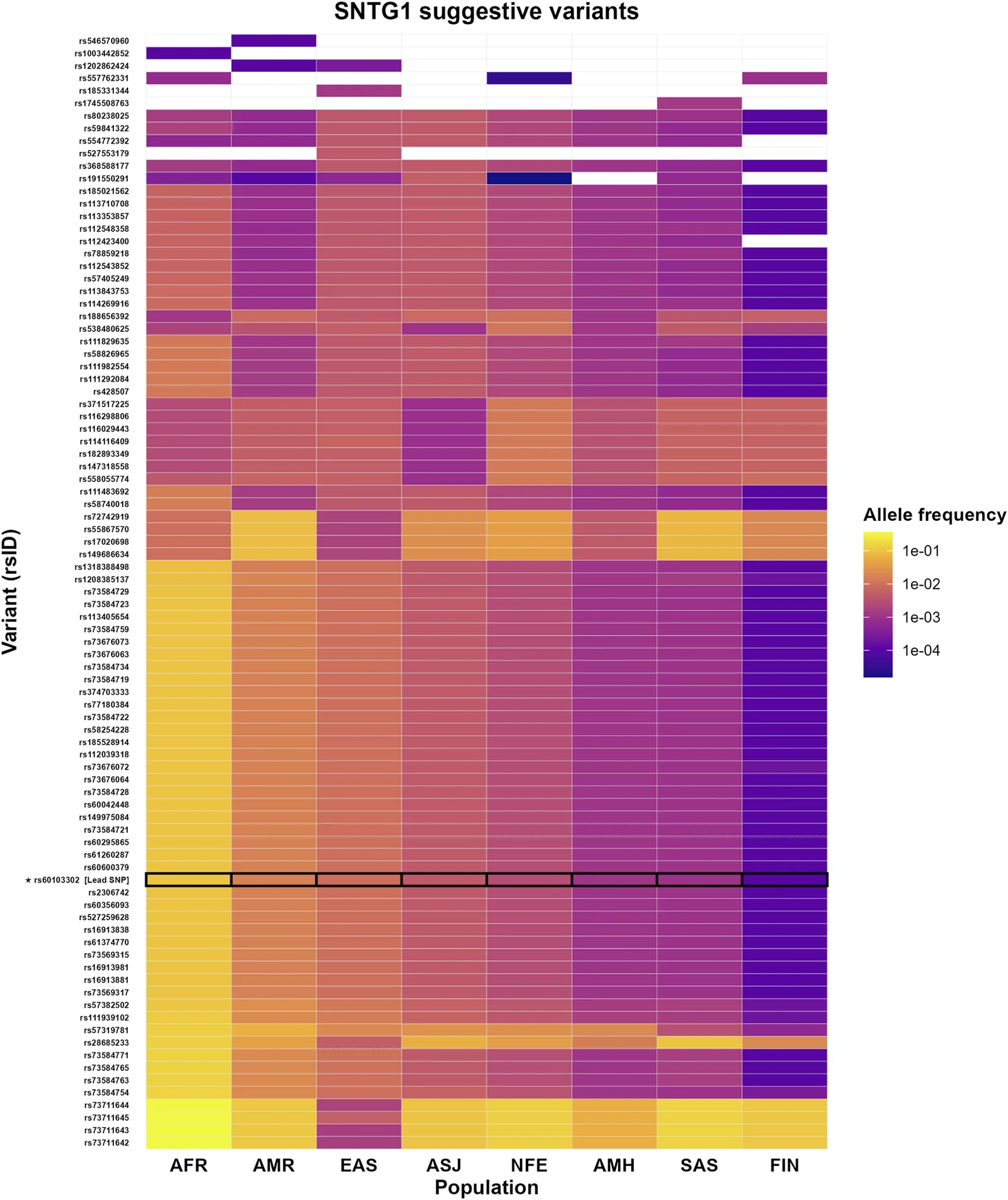
Allele frequency distribution of suggestive variants at the *SNTG1* locus across populations. Heatmap showing allele frequencies of suggestive *SNTG1* variants identified in the SEAD study across diverse ancestral populations from gnomAD v4.1.0. Abbreviations: AFR = African; AMH = Amish; AMR = Admixed American; ASJ = Ashkenazi Jewish; EAS = East Asian; FIN = Finnish; MEA = Middle Eastern; NFE = Non-Finnish European; SAS = South Asian. The lead SNP is highlighted by a black rectangular outline. Allele-frequency data were obtained from Yang et al. (2024). The heatmap was generated by the authors using these published data.

## Functional validation

The researchers went beyond identifying genetic signals by performing functional experiments to investigate how this newly identified variant in *SNTG1* might influence bone formation: specifically, they performed overexpression experiments in mouse preosteoblast cells (MC3T3-E1 cells). Although these experiments were limited to *in vitro* assays in a single cell type, with no *in vivo* and multi-cellular experiment, this attempt to verify gene’s role is notable. Together with replication in other populations incorporating these approaches would strengthen the study, given its aim of elucidating SNTG1’s role in bone metabolism.

## Population specific genotype imputation panels in South and East Asia

Several population-specific genotype imputation panels were developed across Asia, and they showed a potential benefit, particularly for rare variants. The BioBank Japan 1 KG+7K panel improved low-frequency and rare variant imputation relative to TOPMed and enabled the identification of serum uric acid and total cholesterol associations that did not reach genome-wide significance following TOPMed imputation (Flanagan et al., 2024). In South Asia, the GAsP2 reference panel improved coronary artery disease polygenic risk prediction compared with 1KGP-based imputation (Wall et al., 2023). A Southeast Asian-specific (SEA-specific) reference panel also improved the number and quality of imputed variants compared with 1KGP in some global panels in ancestry-matched Orang Asli samples despite its small size (Cengnata et al., 2024). Additionally, the recently developed VN1K reference panel, based on 1011 Kinh Vietnamese individuals, outperformed 1kGP and SG10K in Vietnamese test samples (Tran et al., 2026). These studies demonstrate that population-specific reference haplotypes can improve the recovery of rare and locally enriched variants even when the ancestry-matched panel is smaller than a global panel. Therefore, population-specific and broad regional panels should be considered as complementary, and future imputation studies should benchmark available ancestry-matched reference panels against broader panels, particularly when rare variants are of interest.

## Conclusion

The authors of SEAD did a good job constructing an imputation reference panel for a previously underrepresented Asian population in global reference panels. Nevertheless, substantial gaps in population coverage and the unequal representation of different populations within a reference panel may influence imputation accuracy, particularly for rare and population enriched variants. From Figure 1B, one can see how much more work is expected; Central Asia and Middle East are not well represented in any of the existing resources (at the end of 2025). The recently published Northeast Asian Reference Database, and Indian reference panel represented populations from Northeast Asia and South Asia, respectively. Additionally new population-specific reference resources in Asia, such as the Singapore Precision Health (PRECISE) long-read sequencing project, VN1K for Vietnamese population (Tran et al., 2026), and the First Arab Human Pangenome, could help address this gap. Given that Asia is a vast continent comprising approximately 60% of the world’s population, further studies are essential to enhance coverage.
